# Tumor mutational burden and purity adjustment before and after treatment with temozolomide in 27 paired samples of glioblastoma: a prospective study

**DOI:** 10.1002/1878-0261.13015

**Published:** 2021-06-25

**Authors:** Dorte Schou Nørøxe, Aidan Flynn, Christina Westmose Yde, Olga Østrup, Finn Cilius Nielsen, Jane Skjøth‐Rasmussen, Jannick Brennum, Petra Hamerlik, Joachim Weischenfeldt, Hans Skovgaard Poulsen, Ulrik Lassen

**Affiliations:** ^1^ Department of Radiation Biology Rigshospitalet Copenhagen Denmark; ^2^ Department of Oncology Rigshospitalet Copenhagen Denmark; ^3^ Biotech Research and Innovation Centre (BRIC) University of Copenhagen Denmark; ^4^ Finsen Laboratory Rigshospitalet Copenhagen Denmark; ^5^ Center for Genomic Medicine Rigshospitalet Copenhagen Denmark; ^6^ Department of Neuro Surgery Rigshospitalet Copenhagen Denmark; ^7^ Danish Cancer Society Copenhagen Denmark

**Keywords:** glioblastoma, immune therapy, paired samples, temozolomide, tumor mutational burden, tumor purity

## Abstract

Treatment of glioblastoma (GBM) remains a challenging task, with limited treatment options, none offering a cure. Immune therapy has proven effective across different cancers with remarkable response rates. Tumor mutational burden (TMB) is a marker of response, but technical and methodological differences in TMB estimates have made a proper assessment and comparison challenging. Here, we analyzed a prospective collection of paired samples from 35 patients with newly diagnosed GBM, all of whom were wild‐type (WT) for isocitrate dehydrogenase, before and after treatment with radiotherapy and temozolomide. Seven patients (20%) had O6‐methylguanine‐DNA methyltransferase‐methylated tumors. Six patients (17%) had two relapse surgeries, and tissue from all three surgeries was collected. We found that accurate evaluation of TMB was confounded by high variability in the cancer cell fraction of relapse samples. To ameliorate this, we developed a model to adjust for tumor purity based on the relative density distribution of variant allele frequencies in each primary–relapse pair. Additionally, we examined the mutation spectra of shared and private mutations. After tumor purity adjustment, we found TMB comparison reliable in tumors with tumor purity between 15% and 40%, resulting in 27/35 patients (77.1%). TMB remained unchanged from 0.65 mutations per megabase (Mb) to 0.67/Mb before and after treatment, respectively. Examination of the mutation spectra revealed a dominance of C > T transitions at CpG sites in both shared and relapse‐private mutations, consistent with cytosine deamination and the clock‐like mutational signature 1. We present and apply a cellularity correction approach that enables more accurate assessment of TMB in paired tumor samples. We did not find a significant increase in TMB after correcting for cancer cell fraction. Our study raises significant concerns when determining TMB. Although a small sample size, corrected TMB can have a clinical significance when stratifying patients to experimental treatment, for example, immune checkpoint therapy.

## Graphical abstract

Tumor mutational burden (TMB) is an important clinical marker used to potentially allocate patients for immune therapy. We estimated TMB before and after treatment in 35 paired samples from patients with glioblastoma. Surprisingly, relapse samples presented with a low degree of tumor purity, making intersampling comparison of TMB unreliable. We therefore developed a computational method to adjust for tumor purity in exome sequencing data and found comparison reliable in 27 paired samples. The underexplored role of tumor purity should be included in the development of a standardized method for TMB evaluation in order to stratify clinical data analysis and the evaluation of cancer treatment strategies.

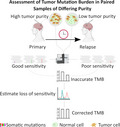

**Abbreviations**
BAM: binary alignment map
bp: base pair
CF: compensation factor
CGC: Copenhagen glioblastoma cohort
COSMIC: catalog of somatic mutations in cancer
cTMB: corrected tumor mutational burden
DC: density‐based compensation factor
EP: estimated tumor purity
FFPE: formalin‐fixed paraffin‐embedded
GBM: glioblastoma
gDNA: genomic DNA
IDH: isocitrate dehydrogenase
IT: immune therapy
Mb: megabase
MGMT: O6‐methylguanine‐DNA methyltransferase
MMR: mismatch repair
NGS: next‐generation sequencing
NSCLC: non‐small‐cell lung cancer
OS: overall survival
PD1i: programmed death‐1 inhibitor
PFS: progression‐free survival
pkVAF: peak variant allele frequency
PS: performance status
RPTMB: reduced‐purity tumor mutational burden
RT: radiotherapy
rTMB: raw tumor mutational burden
SD: standard deviation
SDC: standard deviation‐based compensation factor
SP: spike‐in proportion
tDNA: tumor DNA
TMB: tumor mutational burden
TMZ: temozolomide
TNP: true normal proportion
VAF: variant allele frequency
WES: whole‐exome sequencing
WT: wild‐type

## 1 Introduction

Tumor mutational burden (TMB) is a promising marker of response to immune therapy (IT). In this context, TMB is often defined as the number of nonsynonymous somatic mutations in a tumor sample, and high rates of TMB are associated with genome instability, a hallmark of cancer [1]. A high TMB can cause an increased number of neoantigens that serve to recruit the adaptive immune system and as such provides a potential biomarker for IT response [2]. Good clinical response rates have been shown in TMB‐high tumors like melanoma, non‐small‐cell lung cancer (NSCLC), and mismatch repair (MMR) deficient colon cancer [3, 4, 5, 6]. Mutations in melanoma and NSCLC are mainly caused by exogenous mutagenesis and have a TMB average of 3–400/megabase (Mb; range to more than 1000) [7]. This is opposite of glioblastoma (GBM) [8] with TMB estimates of approximately 1–3/Mb (0–76) [7, 8, 9, 10] and despite a huge unmet medical need, the clinical role of TMB in GBM has not been fully explored. GBM is incurable with a progression‐free survival (PFS) of 7–8 months and a median overall survival (OS) of 16–22 months depending on prognostic and predictive markers [11, 12, 13, 14], including isocitrate dehydrogenase (IDH) status, O^6^‐methylguanine‐DNA methyltransferase (MGMT) promoter methylation status, performance status (PS), use of corticosteroids, and extent of surgery. The incidence of the positive prognostic *IDH*‐mutated GBM is approximately 5% and is more often seen in the malignant transformation from a lower grade glioma to a secondary GBM. Current standard of care for newly diagnosed GBM was introduced in 2005 and consists of maximum‐safe surgical resection, followed by radiotherapy (RT) plus concurrent and adjuvant chemotherapy with temozolomide (TMZ) [15]. TMZ is an alkylating drug and can cause hypermutated phenotypes [16, 17]. OS has not changed significantly since 2005, and new treatment strategies are urgently needed. Response to IT in GBM has been shown in case series based upon mutations in MMR genes [18, 19, 20] making TMB clinically interesting in GBM. Tumors with high TMB are more prevalent in TMZ‐exposed high‐grade gliomas with a prevalence of 3.5% to 17% and is more often seen in *IDH*‐mutated samples [9, 17, 21, 22, 23]. This clonal evolution during TMZ exposure may make the resistant tumor more susceptible to IT. However, a study in recurrent GBM with bevacizumab vs the programmed death‐1 inhibitor (PD1i), nivolumab, showed no difference in OS [24, 25]. The study did not stratify for TMB, but TMB is being analyzed retrospectively. In the present study, we sought to investigate TMB before and after exposure to first‐line treatment in paired samples from 35 patients with newly diagnosed GBM, *IDH*‐WT. We examined the influence of tumor purity on TMB estimates and applied a simple method to perform tumor purity adjustment. This enabled a comparable analysis between tumor samples with vastly different tumor purities, which is especially pertinent in relapse samples since they often have low tumor cellularity, for example, due to infiltration of inflammatory cells following exposure to RT and TMZ.

## 2 Materials and methods

### 2.1 Patients

A total of 35 patients were included from the Copenhagen GBM Cohort (CGC) from February 2016 to August 2018 at Rigshospitalet, Copenhagen. All patients who had relapse surgery performed in this period, and regardless of treatment, were included. All patients were newly diagnosed with GBM based on the 2016 WHO classification [1] and had a second surgical procedure performed due to progression (Table 1). All patients signed an informed consent. Clinical data were noted through patient interviews and medical records, including age at diagnosis, gender, PS, oncologic treatment, PFS, and OS. Date of datalock was 10.03.2019. The project was carried out in accordance with the Declaration of Helsinki and with approval from the Danish National Ethics Committee (journal numbers: H‐3‐2009‐136 and 1707335) and Danish Data Protection Agency (journal numbers: 2014‐41‐2857 and VD‐2018‐204 with I‐suite number: 6447).

**Table 1:** Patient characteristics.

Patients, *N*	35
Female, *N* (%)	10 (28.6)
Age at diagnosis, median (range)	61 (40–80)
ECOG PS, *N* (%)	
After diagnostic surgery	
0	20 (57.1)
1	13 (37.1)
2	2 (5.7)
PS, *N* (%)	
After relapse surgery	
0	11 (31.4)
1	12 (34.3)
2	12 (34.3)
MGMT‐non‐methylated, *N* (%)	28 (80)
IDH‐WT, N (%)	35 (100)
Treatment, *N* (%)	
STUPP^a^	28 (80)
IT (trial)^b^	4 (11.4)
Other^c^	3 (8.6)
Sample preservation, *N* (%)	
Diagnostic surgery	
RNA*later*	25 (71.4)
FFPE	8 (22.9)
Snap‐frozen	2 (5.7)
Sample preservation, *N* (%)	
Relapse surgery	
RNA*later*	19 (54.3)
FFPE	16 (45.7)
Snap‐frozen	0 (0)
PFS, months (median)	
Diagnostic surgery	7.5
Relapse surgery	5.5
OS, months (median)	
Diagnostic surgery	16.2
Relapse surgery	8.9

^a^ RT with 60 Gy/30F concurrent with TMZ followed by adjuvant TMZ.

^b^ RT/TMZ plus a PD1i in a trial. One patient received RT/TMZ plus PD1i or placebo.

^c^ 30 Gy/10F or 60 Gy/30F.

### 2.2 Collection of tissue

The methods used for collection of tissue and for whole‐exome sequencing (WES) have been described elsewhere [26]. Three representative tissue biopsies collected at surgery were immediately preserved in *RNA*later for optimal DNA and RNA purification. In case of insufficient amount of tissue, we used supplemental tissue that was either snap‐frozen or formalin‐fixed paraffin‐embedded (FFPE). Patients further delivered a blood sample for germline subtraction in order to identify somatic variants only. If suspicion of disease promoting inherited variants was raised, we would perform further analyses and contact the patient. However, we did not identify any suspected inherited variants.

### 2.3 Whole‐exome sequencing

Whole‐exome sequencing was performed using DNA from tissue and blood. DNA from tumor samples [tumor DNA (tDNA)] was extracted using the AllPrep DNA/RNA purification kit and the QIACube workstation (Qiagen, Hilden, Germany) according to manufacturer's instructions. Genomic DNA from whole blood samples (gDNA) was isolated using the liquid handling automated station (Tecan, Männedorf, Switzerland). Purified DNA was quantified using the Qubit instrument (Life Technologies, Thermo Fisher Scientific, Waltham, MA, USA). gDNA (200 ng) was fragmented to 300 base pair (bp) using Covaris S2 (Agilent, Santa Clara, CA, USA), and adaptor ligation was performed using KAPA HTP Library Preparation Kit (Roche, Basel, Switzerland). Exomes were enriched with SureSelectXT Clinical Research Exome kit (Agilent). Paired‐end sequencing (2 × 100 bp or 2 × 150 bp) was performed to gain an average coverage of 50–100×, using the HiSeq 2500 or NextSeq 500 platforms from Illumina. Raw sequencing data were processed using CASAVA‐1.8.2. Reads were aligned to the human reference genome (hg19/GRCh37) using bwa‐mem (v0.7.10, Cambridge, UK). Somatic variant calling was performed using MuTect (v1.1.7) [27]; a high‐confidence call set was established by removing frequently miscalled sites and variants with an allele frequency below 10% in the tDNA. Somatic variants were identified by excluding variants found in blood WES data from the patient and further analyzed using Ingenuity Variant Analysis (Qiagen). To assess the stability of tumor mutation burden between versions of the MuTect variant caller and for comparison with existing literature, somatic variant calling was also performed using MuTect2 from the Genome Analysis Toolkit (gatk, v4.1.6.0, Cambridge, MA, USA) and high‐confidence variants were selected as described above.

### 2.4 Simulated low‐purity data

Exome sequencing data from routine diagnostic sequencing at Rigshospitalet of 38 high purity GBM tumors, including six pairs, were used to construct artificial reduced‐purity data. SAMtools subsampling was used to combine reads from each tumor with reads from the matched normal such that the normal reads contributed 40, 60, 70, or 80 percent of a total 100 million reads. MuTect was used to perform variant calling on the resulting binary alignment map (BAM) files.

### 2.5 Mutation burden purity scaling

To compensate for differences in sensitivity arising from tumor purity, we computed a compensation factor (CF) between samples with differing purity. First, a density distribution was computed for the variant allele frequencies (VAFs) of each sample using a Gaussian kernel. Peak finding was performed using the *peak* function from *splus2r* package for r (Vienna, Austria). The peak representing clonal heterozygous mutations was determined by selecting the peak at the greatest VAF (pkVAF) where the magnitude of the peak was at least one‐third of the highest magnitude peak present. The difference in pkVAF values between paired samples was calculated, and the value was subtracted from VAF of all variants in the sample with the greater pkVAF. Any variants with a negative VAF were considered to be below the artificial detection threshold and removed to create a reduced‐purity TMB (RPTMB). The density‐based CF (DC) was determined by subtracting the RPTMB from the raw TMB (rTMB) and dividing by the RPTMB.To accommodate samples with significantly different density distribution or large subclonal populations, a second CF was determined based on the number of variants close to the pkVAF. The standard deviation (SD) for the VAFs in each sample was determined, and the number of variants within half a SD of the pkVAF for each density distribution was calculated. The count for the lower purity sample was subtracted from that of the higher purity sample, and the result was divided by the count of the lower purity sample to generate the SD‐based CF (SDC). Samples with a similar purity will experience commensurate loss of sensitivity necessitating minimal correction compared to those with a large purity differential. To offset this, a weighting system based on relative sample purity was developed. The weight was determined as the absolute value of one minus the result of dividing the higher sample purity by the lower sample purity, with an upper limit of one {W|0 < *W *> 1} = |1 ‐( P1/P2)|. The final CF was determined as the average of the SDC and DC CFs multiplied by the purity differential weight (*W*). The corrected TMB (cTMB) for the lower purity sample was determined as the rTMB plus the CF times the rTMB. cTMB = rTMB + (CF*rTMB). In cases where the calculated CF was negative, no adjustment was made.

### 2.6 Mutation spectra analyses

Mutational contexts were extracted using the YAPSA package (v1.8) [28] for the r statistical framework. Base composition was based on the UCSC HG19 genome *via* the bsgenome (BSgenome.Hsapiens.UCSC.hg19, Seattle, DC, USA) package for R. Mutational signature analysis was performed using the SigFit package [29]. Signature fitting was performed with catalog of somatic mutations in cancer (COSMIC) version 3 SBS signatures 1, 3, 5, 8, 11, 15, 16, 30, and 40 using the following parameters: iter = 2000, warmup = 1000, chains = 1, seed = 1756, hpd_prob = 0.9). Signatures with a lower highest posterior density bound > 0.01 were considered high confidence.

### 2.7 Purity estimation with Sequenza

Matched tumor and normal sequencing reads in binary alignment format were processed using the Sequenza package (v3.0) for the r statistical framework with 50 kilobase windows. The optimal purity and ploidy solution were automatically selected by the Sequenza algorithm.

## 3 Results

### 3.1 Clinical data

**Fig. 1:**
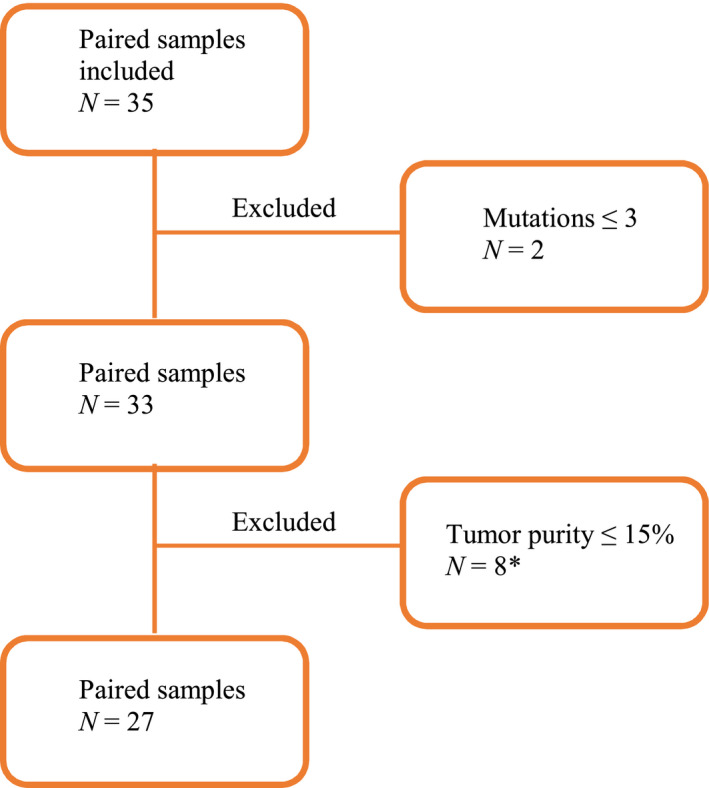
Consort diagram of included paired samples. *N* = 35. Two samples were excluded upfront due to a mutation count ≤ 3 in the relapse sample. Further eight samples were excluded due to a tumor purity ≤ 15%. *Two of the excluded samples came from two patients with three surgeries. Each patient had a reliable diagnostic sample and another reliable sample from the second relapse and first relapse, respectively. Hence, a total of 10 samples from eight patients were excluded, leaving 27 patients with paired samples reliable for further analyses.

A total of 35 patients were included. Seven patients (20%) had MGMT promoter methylated tumors. Six patients (17%) had three surgeries performed due to a second relapse. For patient characteristics, see Table 1. Two patients had ≤ 3 mutations in the relapse sample and were categorized as not having progression. This decision was supported by lack of vital tumor cells upon the histopathological examination and, ultimately, by the clinical decision of not treating as progression; one patient continued the adjuvant TMZ after the relapse surgery and the other patient continued in a follow‐up program with no treatment. Both patients were without evidence of progression at time of datalock, living +13 and +22 months, respectively, from diagnosis. A total of eight samples were excluded due to low tumor content ≤ 15%, and all were relapse samples. Two of the excluded samples were from patients with three surgeries. The patients had two reliable samples that could be used for TMB comparison, from diagnostic surgery and the second relapse (RGHB022) and first relapse (RGHB027), respectively. Hence, a total of 10 samples were excluded from 8 patients, leaving 27 patients with paired samples for further analyses (Fig. 1 and Fig. S1).

### 3.2 Relapse specimens exhibit lower tumor content

**Fig. 2:**
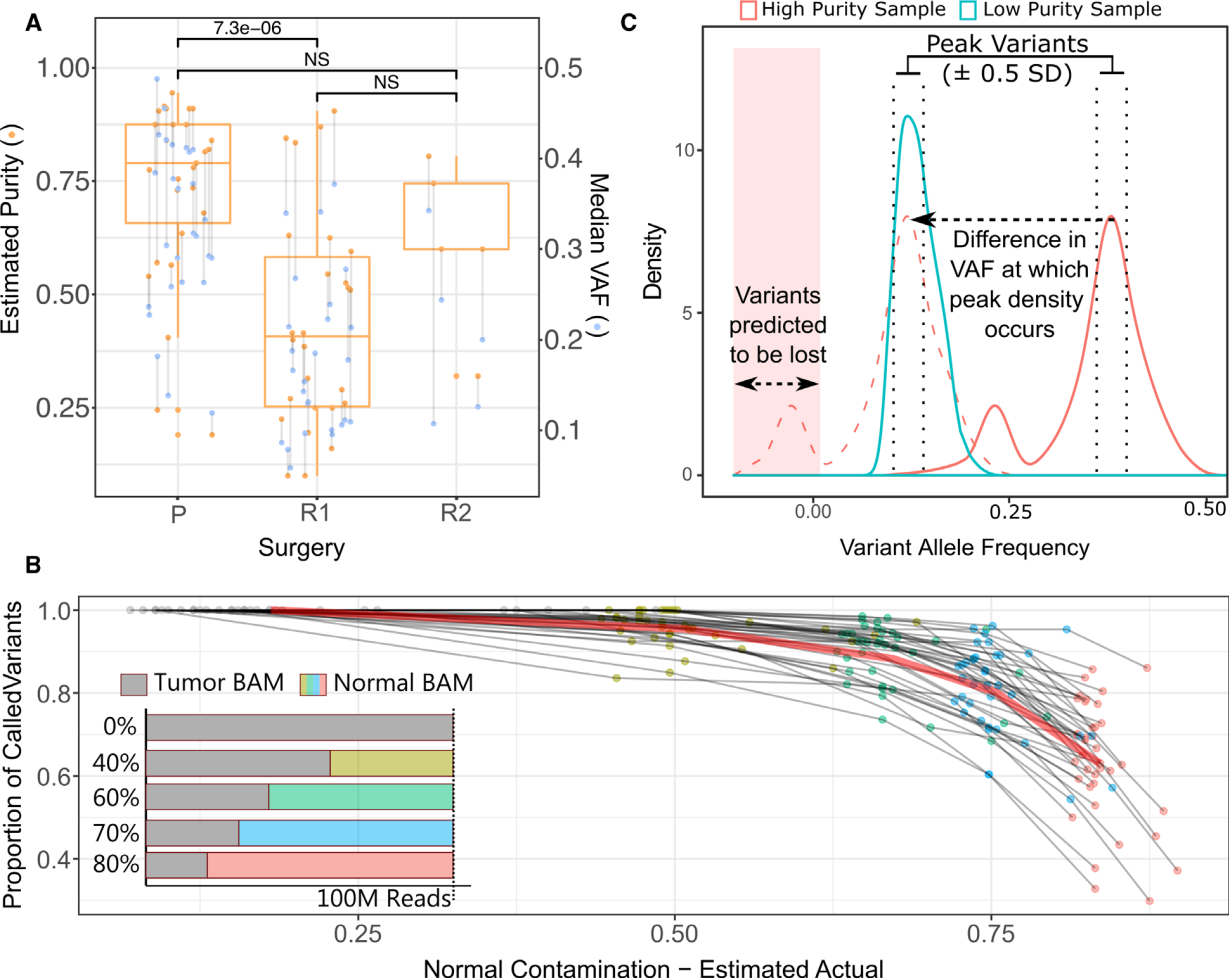
Correcting for the adverse effect of tumor purity on variant calling sensitivity. (A) Estimates of tumor cellularity using the Sequenza algorithm (orange dots and box plots, left axis). The box plot shows the 25%, 50%/ median, and 75% quantile. The median somatic VAF (blue dots, right axis) is shown as a surrogate marker of tumor cellularity. Tumor samples from the same patient are joined by a vertical line. Surgical time points are marked as primary (P), first relapse (R1), and second relapse (R2). The difference in the mean VAF between surgical time points was assessed using a paired, two‐tailed *t*‐test, and comparisons are annotated either as not significant (NS) or with their resulting *P*‐value. (B) The effect of increasing contamination with normal tissue on variant calling sensitivity was assessed by in silico serial dilution of tumor sequencing data with matched germline data for 38 GBM samples. The impact was assessed at 40% (gold dots), 60% (green dots), 70% (blue dots), and 80% (red dots) normal contamination (see inset). The estimated real‐world normal proportion was calculated as the product of the Sequenza cellularity estimates and the in silico dilution (*x*‐axis). Variant recovery is expressed as a proportion of undiluted tumor data. The median proportion of variants recovered in all samples is indicated by the red line. (C) Visual schematic illustrating the approach used to compensate for differences in tumor purity when assessing tumor mutation burden. Briefly, the peak density of VAFs was computed for each sample. The difference between the VAF at which the highest density occurred in each sample was subtracted from the VAF of each variant in the sample with the greater peak VAF. After subtraction, variants with a negative adjusted VAF were removed and the difference of the pre‐ and postfiltration variant counts was divided by the postfiltration count to compute a scaling factor. A second scaling factor was computed based on the ratio of the number of variants with a VAF within 0.5 SD of the respective peak for each sample.

Accurate assessment of TMB is integral for identification of those who may benefit from immune checkpoint therapies. In our initial analysis of TMB, it became apparent that many of the relapse specimens exhibited lower TMB than their matched primary. This result was counterintuitive given the generally accepted model of mutation accrual throughout the life of a tumor. To clarify this observation, we examined the median VAF of the somatic mutations detected in each specimen. The VAF provides a surrogate marker for the proportion of cells harboring a given mutation. In a pure specimen, a clonal mutation (one present in all tumor cells) such as a core driver or early passenger mutation will have a VAF of 0.5 (within diploid regions). Analysis of the median VAF revealed that on average, the relapse samples (mean: 0.15, range: 0.04–0.3) had a considerably lower median VAF than those from diagnostic specimens (mean: 0.32, range: 0.10–0.48; Fig. 2A, blue). We corroborated this finding using the Sequenza algorithm to estimate tumor purity (Fig. 2A, orange).

### 3.3 Adjusting TMB for tumor purity

Next‐generation sequencing (NGS) is a stochastic process where short DNA segments are selected at random from a population for sequencing. The sensitivity of this process to detect mutations is a function of the amount of sampling (sequencing depth) and the prevalence of the mutation within the DNA population. The latter itself is dependent on two factors: the first being that of clonality, where a mutation can be present in a subpopulation of the tumor cells, and the second being the presence of nontumor cells within a surgical specimen. In both these cases, the presence of normal (nonmutant) DNA serves to dilute the mutant DNA reducing the likelihood of detection. In order to better understand the relationship between the presence of normal (nontumor) contamination, we performed a series of in silico dilutions by spiking variable amounts of normal sequencing data into data from high purity tumor specimens (Fig. 2B—inset). Spike‐in was performed to produce BAM files with 100 million reads containing 40%, 60%, 70%, and 80% normal data. As the tumor data used to produce the BAM files already contained a portion of normal reads, the estimated true normal proportion (TNP), following spike‐in was computed using the estimated tumor purity (EP) of the original specimen and the spike‐in proportion (SP) as: TNP = 1 − (EP × (1 − SP)). A total of 38 independent tumors were analyzed to ensure representation of a diverse set of mutational profiles. Mutation calling was then performed on the spike‐in BAM files using the MuTect algorithm, and the proportion of variants detected compared to the unadulterated BAM was computed for each contamination level (Fig. 2B). The analysis revealed a nonlinear relationship between increasing normal contamination and loss in sensitivity with an average of 96%, 88%, 80%, and 64% of variants still detectable at 40%, 60%, 70%, and 80% normal spike‐in, respectively. Based on this result, we excluded tumors with < 15% tumor content from further analysis. Using this cutoff, we excluded 8/33 paired samples (24.2%) and all excluded samples were from relapse surgery (Figs S1 and S2).

### 3.4 Compensating for sensitivity loss

To facilitate TMB comparisons between samples of varying purity, we attempted to model the loss of sensitivity in the less pure samples. Initially, a density distribution of VAFs was computed for each sample in the comparison. The peak representing clonal heterozygous mutations was assumed to be that with the greatest VAF where the density was at least two‐thirds that of the highest density peak. This helped to prevent incorrect peak assignment in cases that had a small number of mutations in regions of chromosomal loss or copy‐neutral loss of heterozygosity. To approximate the loss of sensitivity in the lower purity sample, the difference in the peak VAF between samples was computed and subtracted from the frequency of each variant in the higher purity sample. Following adjustment, variants with a frequency below zero were considered below detection threshold and excluded (Fig. 2C). The ratio of mutations before and after filtering was used as a primary adjustment factor. Depending on the clonal and subclonal composition of a sample, the density distribution of VAFs can vary considerably even between patient matched samples. The ‘sliding density distribution’ approach described above is vulnerable to such differences. To ameliorate this, a secondary adjustment factor was calculated by determining the ratio of the number of mutations with a VAF that falls within ± 0.5 SDs of the peak VAF in each sample. This metric is more stable as it relies on the more easily detectable clonal mutations; however, it does not capture the complexity of the whole distribution. Finally, to account for the nonlinear falloff in sensitivity, a weight was applied to the average of the two scaling parameters calculated above.

### 3.5 Assessment of the TMB correction model

**Fig. 3:**
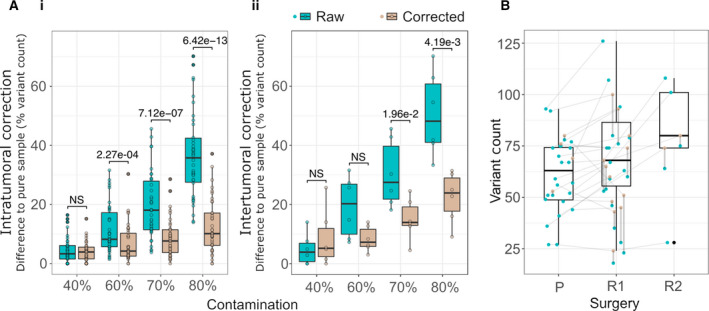
Assessment of purity adjustment on simulated and patient data. (A) Difference in variant count relative to the pure sample for each level of simulated impurity before (green) and after (brown) the correction model is applied. The pure data were tested using (i) intrasample correction and (ii) intersample correction. Whether mean error was reduced after correction was assessed by way of a paired, one‐tailed *t*‐test, and comparisons are annotated either as not significant (NS) or with their resulting *P*‐value. (B) TMB of GBM samples at presentation (P), first relapse (R1), and second relapse (R2). Uncorrected (green) and corrected (brown) TMB values are shown for each specimen and joined by a vertical line. Specimens from a single patient are connected with a horizontal line. A summary of the cTMB values is shown as boxplots. The box plot shows the 25%, 50%/median, and 75% quantile.

To assess the accuracy of the TMB correction model, we applied the model to the in silico serial dilution series. Initially, we utilized the undiluted data for each sample to correct the 40%, 60%, 70%, and 80% normal spike‐in data for the same sample. As an assessment metric, we calculated the difference in the number of mutations detected at each spike‐in level compared to the undiluted data expressed as a percentage of the number of mutations detected in the undiluted data. Calculating this metric for pre‐ and postcorrection variant counts demonstrated that the correction provided a reduction in error by 4.7%, 11%, and 23% for the 60%, 70%, and 80% spike‐in data, respectively, and an increase in error by 0.5% for the 40% spike‐in data (Fig. 3A). In a real‐world scenario, two independent samples, even those from a single patient, are likely to have different VAF density distributions. To assess how the method would perform under these conditions, we used undiluted data from a primary paired with a dilution series from the matched relapse for six cases with sufficiently pure relapse samples. Cross‐sample correction demonstrated a reduction in error by 10.8%, 15.3%, and 28.1% for the 60%, 70%, and 80% spike‐in data and an increase in error by 3.7% in the 40% data. In both scenarios, correction at the 40% contamination increased the error margin, suggesting that samples with > 50% tumor content do not benefit from TMB correction. However, correction can provide more accurate TMB estimates in samples with a total tumor percentage below 50% where the partnered sample is of higher purity. As previously mentioned, samples below 15% tumor content are likely below the threshold at which accurate TMB can be ascertained with or without correction.

### 3.6 Tumor mutational burden and signature analyses before and after treatment

**Fig. 4:**
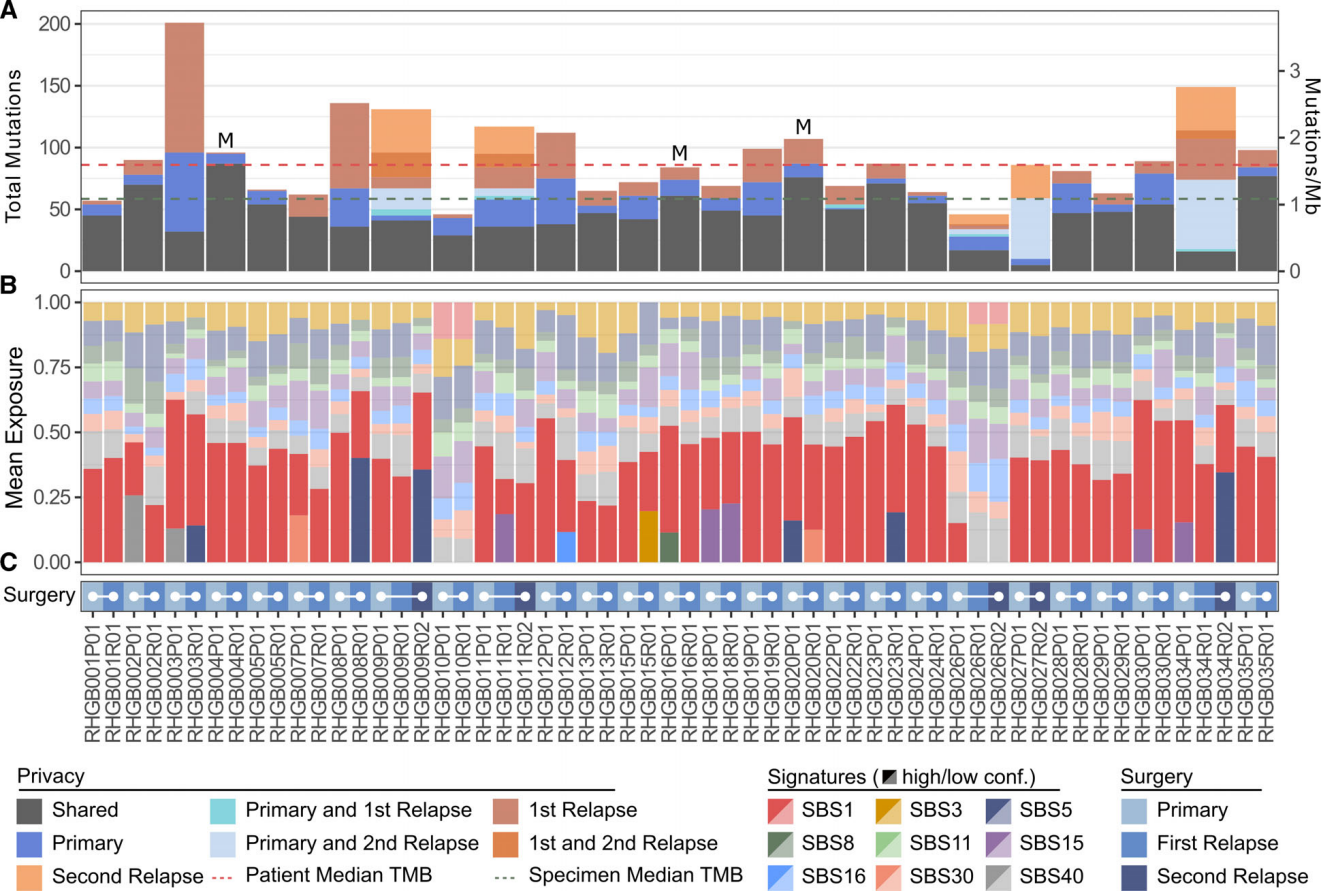
(A) The total mutation burden for each patient was calculated after mutation calling with the MuTect algorithm. Mutations called in any patient specimen were manually assessed in each specimen from the respective patient to determine the presence or absence of the mutation. Mutations found in all specimens from a given patient are annotated as shared; otherwise, they are annotated with the surgical time point(s) in which they were present (colored bars). The median number of mutations per sample (green) and per patient (red) is indicated as dashed lines. M = MGMT methylation. (B) Mutational signature analysis was performed with the SigFig algorithm using signatures previously described in GBM (SBS1, SBS3, SBS5, SBS8, SBS11, SBS15, SBS16, SBS30, and SBS40). High (dark colors) and low (light colors) confidence signatures were defined as those with a 90% highest posterior density interval above and below 0.01, respectively. (C) Color chart indicating the surgical time point of the data in panel (B). Samples from the same patient are joined by a solid white line. *N* (paired samples) = 27.

We next performed a comparative analysis of the TMB between the paired samples and identified the number of shared and private mutations in each patient (Fig. 4A). An adjusted median TMB before and after treatment was stable at 0.65 (range: 0.24–1.02) vs 0.67 (range: 0.20–1.38), respectively. The majority of mutations were shared between the primary and relapse sample; however, most of the relapse samples presented with at least one relapse‐specific mutation. All patients with three surgical procedures presented with mutations private for the relapse, suggesting ongoing mutagenesis. We next investigated single‐base substitution signatures using a curated set of mutation signatures previously published in GBM (SBS 1, 3, 5, 8,11, 15, 16, 30, and 40) made available by the COSMIC (Fig. 4B). Signature 1 (SBS1), which is associated with spontaneous deamination of 5‐methylcytosine and generally considered a consequence of cellular aging, was the most prevalent signature across the cohort. The DNA MMR deficiency signature SBS15 was found in five cases; however, none of these samples had mutations in MMR pathway genes or elevated TMB. We did not find evidence of SBS11, associated with TMZ exposure; however, we also did not see evidence of TMZ‐induced hypermutation profiles.

### 3.7 Treatment did not induce hypermutation

In the TMZ‐exposed patients (*N* = 20), TMB remained the same before and after treatment with a mean TMB of 0.69 to 0.68 (range 0.24–1.02 to 0.33–1.38, *P* = 0.79 using a paired *t*‐test), respectively. Three patients received IT in a trial and had an unchanged mean TMB before and after treatment of 0.52 to 0.51 (range 0.44–0.58 to 0.33–0.75, *P* = 0.95), respectively. The remaining three patients received RT only with no change in mean TMB from 0.66 to 0.67 (range 0.35–0.82 to 0.42–0.91, *P* = 0.97), respectively. One patient participated in a trial with RT/TMZ plus nivolumab or placebo. Since we did not know if the patient had received IT or not, we chose to exclude the patient in the above analyses.

## 4 Discussion

Here, we present results from the CGC with paired samples in a vulnerable patient cohort where tissue is difficult to obtain due to the invasive procedure of brain surgery. The inclusion period extended over 2½ year, and the median PFS was 7.5 months; accordingly, the actual inclusion period for the relapse sampling was < 2 years. At our institution, we perform relapse surgery on approximately 30 patients per year, so this study represents an inclusion of 60–70% of eligible patients. Previous studies on paired samples in GBM have been with sample sizes of < 40 and, to our knowledge, have been on archival tissue [30]. This illustrates the complexity in obtaining paired samples in GBM patients. To address these challenges and increase availability of datasets from paired samples, the international GLASS consortium has been initiated with the aim of generating longitudinal genomic and molecular data in *IDH*‐WT, *IDH*‐mutant, and 1p/19q codeleted tumors [9, 30]. Upfront, we had to exclude two samples from patients not having true progression and hence limited mutations in the samples. They illustrate the dilemma with pseudoprogression subsequent to chemoradiation with TMZ.

### 4.1 Contamination with normal tissue complicates paired TMB comparison

The digital nature of NGS technologies provides excellent sensitivity for detecting mutations in mixed populations of cells. Despite this, these technologies are still vulnerable to the effects of sample purity and preservation and excessive contamination with normal tissue will adversely affect the sensitivity of variant calling algorithms [31]. Tumor heterogeneity might also affect results as clonal mutations are present in majority of tumor cells, but it cannot be excluded that subclonal mutations are present in only a subset of the tumor cells. However, the role of tumor purity is still unchallenged. Majority of the relapse samples had low tumor purity, which affects TMB estimates and can render both intrapatient and between‐studies comparisons as unreliable. Therefore, we developed a method to adjust for tumor purity by adjusting the density distribution of the VAFs in the purer sample to match that of the less pure sample. Our assumption was that in normalizing the densities, we could simulate the rate at which information was lost from the less pure sample through normal cell contamination. We then used the ratio of the original counts in the pure sample to the ‘postnormalization’ counts to produce a scaling factor. By multiplying the scaling factor, we obtained an approximation of what the counts might have been in the less pure sample if it had higher tumor content. We found that the purity‐based correction became unstable at tumor purities below 15%. Based on our results, we suggest using a correction scale to compensate for low tumor purity if the tumor purity is between 15% and 40%. Since our purity adjustment method is based on VAF estimates from base substitutions, we do not see any biases related to specific molecular subgroups, for example, MGMT status. However, this should be investigated in future studies. Another dilemma in TMB evaluation and genomic testing is the turn‐around time. This is essential in a clinical setting, as a GBM patient cannot afford to wait too long to start treatment. At our institution, we have used WES for newly diagnosed GBM since 2016 with results ready for second‐line treatment and we are now implementing whole‐genome sequencing for newly diagnosed patients with a clinically relevant turn‐around time for both first‐line and second‐line treatments.

### 4.2 Hypermutation and signature analysis

We did not identify TMZ‐induced hypermutation. This was unexpected since hypermutation has been found in up to 17% of TMZ‐exposed relapse samples [17, 32]. This may partly be explained by our small patient cohort and the low prevalence of MGMT methylation. A correlation between MGMT methylation and MMR‐deficient GBM has been identified and as our cohort of 27 patients with paired samples reliable for TMB comparison included only three patients with MGMT methylation, this might be another explanation for lack of identification of hypermutated samples. Patient RHGBM003 had the highest increase in TMB after treatment from 0.96/Mb to 1.38/Mb. Even so, the increase could not qualify for development of a hypermutated phenotype. Mutational signature analysis has been used across a wide range of cancers to explore underlying mutational processes driving tumor evolution. Opposite to what we expected, signature analysis did not reveal MMR signatures nor development of the TMZ‐signature SBS11. However, it is well established that the sensitivity of mutational signature analyses is highly dependent on larger sample cohorts and we note that the low number of overall mutations may decrease the sensitivity to less frequent mutational signatures.

### 4.3 Comparison of TMB between cancers

When comparing to other cancer types, our study‐specific median TMB before and after treatment of 0.65 vs 0.67 was lower than previously reported in GBM and most other tumor types. Previous studies have reported TMBs from 0 to 76 in GBM [7, 8, 33]. However, it is difficult to compare TMB across cancers due to different etiologies. Therefore, inclusion criteria to experimental histology‐agnostic trials based on TMB score alone can make inclusion of GBM patients difficult and TMB scoring should preferably be compared to the same disease entity. Comparison in brain cancer alone can even be challenging as studies have included high‐grade, low–grade, and pediatric gliomas, primary and relapse samples with limited data on prior treatment, unknown tumor purity estimation, different NGS and data processing methods applied, and lack of validated assays [4, 5, 8, 19, 21, 22, 23, 34, 35, 36, 37, 38]. A recent large‐scale study of 288 glioma patients with paired samples included 134 *IDH*‐WT tumors, thereby resembling our cohort. They identified a TMB of 2.85, using MuTect2 [9]. We analyzed our data using MuTect1 but for the matter of comparison, we pooled all mutations together per patient and found a combined median TMB/Mb of 0.96 (MuTect1) vs 1.05 (MuTect2; Table S1). One explanation to our lower TMB might be that we have used nonsynonymous mutations to report TMB instead of reporting all mutations in each sample. Nevertheless, this illustrates the complexity in TMB estimation and until standardized methods have been developed and accepted, no cutoff value for, for example, TMB high can be defined and thus will always need to be interpreted in the study‐specific cohort. Whether or not hypermutation caused by somatic vs germline mutations or by treatment like TMZ is comparable remains unclear. However, it has been shown that shared mutations represent the clones responsible for the positive selection driving tumorigenesis [9] thereby making identification of specific tumor mutations important instead of the total, unselected TMB. This contributes to the fact that results from relapse studies cannot be directly translated to newly diagnosed patients.

### 4.4 TMB as a predictive marker to IT in GBM

The use of TMB as a predictive marker of response to IT has great potential. However, it will be relevant for only a minority of GBM patients as illustrated by the results in our study; none had a TMB score allowing for IT according to present approved inclusion criteria and none developed a hypermutation after treatment. Since hypermutation is greater in relapsed TMZ‐exposed patients as compared to newly diagnosed patients, it would be expected that IT could have an important role for relapse patients. However, the number of neoantigens vs nonimmunologic changes seems to remain the same before and after treatment, indicating lack of a beneficial effect of IT in relapsing GBM [9]. The first phase III study in relapse samples, The CheckMate 209‐143, investigated the PD1i nivolumab vs bevacizumab [39]. Results did not show superiority to nivolumab. However, it was found that duration of response was longer in the nivolumab‐treated group and that patients with MGMT‐methylated tumors and no corticosteroid dose at baseline had a longer median OS. This suggests that IT is relevant for a small subgroup [39, 40] and the predictive potential of *IDH* and MGMT status for IT should be evaluated in future studies. We could not evaluate MGMT promoter methylation status and IT in our study due to the low number of patients and the low incidence of MGMT promoter methylation of 20%. This is lower than the 40–50% that has been reported in other large studies [26, 41, 42], including the CGC cohort, that our patients were included from. The CGC had an incidence of MGMT promoter methylation of 44.4%, and we explain the lower incidence in the present study by the low number of patients. Recently published press releases from two large phase III trials with nivolumab [43, 44] to newly diagnosed GBM did not show a better OS in the nivolumab‐treated patients, but subgroup analyses are being performed and TMB was included as a secondary endpoint in the CA‐548 study. Results are awaited. The predictive role of TMB cannot stand alone though, since response to IT has also been shown in melanoma, GBM and NSCLC tumors with low TMB [4, 5, 45]. Other factors for response to IT are age, tumor‐infiltrating lymphocytes, PD‐L1 expression, mutations and expression of DNA repair genes [46, 47]. Furthermore, studies with IT in TMB‐low tumors like prostate and pancreatic cancer have been negative [8], but new studies with IT in these cancers are active, underlining that TMB is not regarded as an exclusive factor of response to IT.

## 5 Conclusion

TMB is an important clinical marker to potentially allocate patients for IT, preferably in clinical trials. We found that TMB estimation was possible in all paired samples from 35 included patients. However, relapse samples presented with a low degree of tumor purity, making intersampling comparison of TMB unreliable. Therefore, we developed a method to adjust for tumor purity and found 27 paired samples reliable for TMB comparison. Signature SBS1 was the most prominent signature, associated with cellular aging. We did not find a signature SBS11, associated with TMZ exposure, nor did we find hypermutation after treatment. A standardized method for TMB evaluation is greatly needed, and the undiscovered role of tumor purity should be included in this development. Not until a standardized, international accepted assay has been developed, can international accepted cutoff values be identified and ultimately used for clinical trials and evaluation of treatment.

## Conflict of interest

The authors declare no conflict of interest.

## Author contributions

DSN, AF, JW, HSP, and UL conceived the study. DSN included the patients and designed the clinical database. JSR and JB applied the specimens, PH headed the preparation of tissue. FCN, CWY, and OØ headed the tissue purification and the logistic of tissue handling. AF performed the TMB analyses and the purity adjustment analyses. DSN and AF wrote the manuscript. JW, UL, and HSP performed critical review of the manuscript and all authors have read and approved of the manuscript.

### Peer Review

The peer review history for this article is available at https://publons.com/publon/10.1002/1878‐0261.13015.

## Supporting information


**Fig. S1**. Density analysis of the VAFs of each tumor.
**Fig. S2**. Comparison of VAFs for variants found across paired specimens in cases with primary and a single or paired relapse.
**Table S1**. TMB/Mb for MuTect1 vs MuTect2.

## Data Availability

An approval for publication of the database was not included in the consent form from the Danish Data Protection Agency. Therefore, data from the CGC are not publicly available but part of the database can be made available upon request to the corresponding author.
