# Supplementary material for: Tumor mutational burden and purity adjustment before and after treatment with temozolomide in 27 paired samples of glioblastoma: a prospective study
Source: Mol Oncol. 2021 Jun 25;16(1):206–18. doi: 10.1002/1878-0261.13015 (PMC8732341; doi:10.1002/1878-0261.13015)
Supplement: Supplementary file 1 — Fig. S1. Density analysis of the VAFs of each tumor. Fig. S2. Comparison of VAFs for variants found across paired specimens in cases with primary and a single or paired relapse. Table S1. TMB/Mb for MuTect1 vs MuTect2. [file MOL2-16-206-s001.docx]

SUPPLEMENTARY


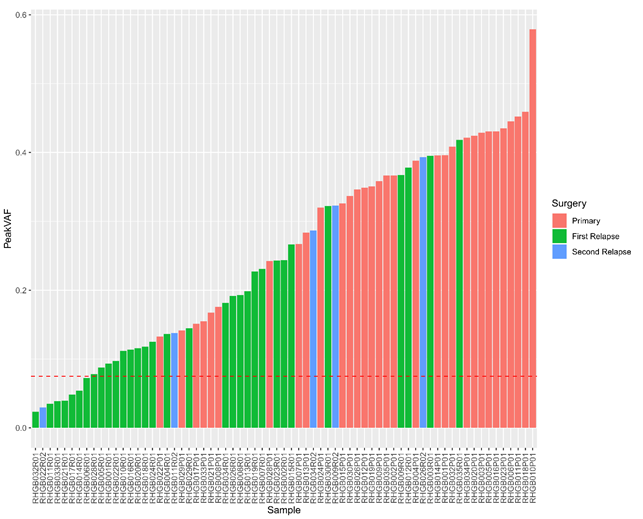


Supplementary figure SF1. Density analysis of the variant allele frequencies of each tumor was used to determine the allele frequency representing clonal variants within each sample. This value was used as a surrogate for tumor purity and a threshold (0.075, red line) was used to exclude samples that were considered unreliable.


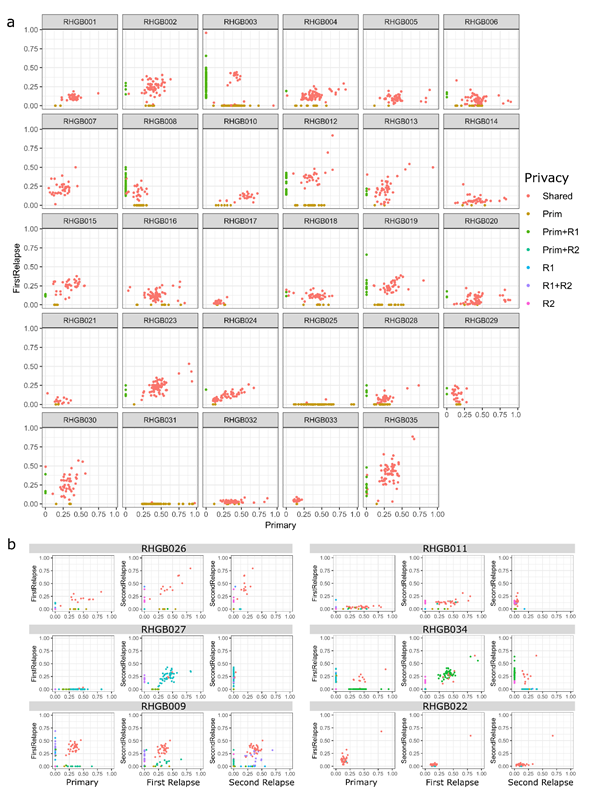


Supplementary figure SF2. Comparison of variant allele frequencies (VAFs) for variants found across paired specimens in cases with primary and a single (a) or (b) paired relapse. Colors indicate the privacy status of the variants as found in the primary (Prim), first relapse (R1) or second relapse (R2).

|  | TMB/Mb | |
| --- | --- | --- |
|  | Mutect1 | Mutect2 |
| PairedSet | Total | Total |
| RHGB001 | 0.53 | 0.45 |
| RHGB002 | 1.24 | 1.29 |
| RHGB003 | 2.15 | 2.29 |
| RHGB004 | 1.00 | 1.11 |
| RHGB005 | 0.64 | 0.60 |
| RHGB007 | 0.69 | 0.58 |
| RHGB008 | 1.49 | 1.58 |
| RHGB009 | 1.45 | 1.31 |
| RHGB010 | 0.51 | 0.45 |
| RHGB011 | 1.16 | 3.15 |
| RHGB012 | 1.35 | 1.20 |
| RHGB013 | 0.82 | 0.76 |
| RHGB015 | 0.67 | 2.00 |
| RHGB016 | 0.85 | 1.05 |
| RHGB018 | 0.80 | 0.73 |
| RHGB019 | 1.16 | 1.33 |
| RHGB020 | 1.04 | 0.98 |
| RHGB022 | 0.82 | 0.69 |
| RHGB023 | 1.00 | 1.04 |
| RHGB024 | 0.71 | 0.85 |
| RHGB026 | 0.53 | 0.45 |
| RHGB027 | 0.98 | 1.09 |
| RHGB028 | 0.93 | 0.95 |
| RHGB029 | 0.67 | 0.75 |
| RHGB030 | 0.96 | 1.87 |
| RHGB034 | 1.62 | 1.58 |
| RHGB035 | 1.05 | 1.18 |

Supplementary table ST1: Tumor mutational burden/Megabase (TMB/Mb) for Mutect1 vs Mutect2. All mutations found across all samples for each patient were pooled together into a complete mutation set. *N* (paired samples) = 27.
